# Detoxification of 1,1,2-Trichloroethane to Ethene by *Desulfitobacterium* and Identification of Its Functional Reductase Gene

**DOI:** 10.1371/journal.pone.0119507

**Published:** 2015-04-02

**Authors:** Siyan Zhao, Chang Ding, Jianzhong He

**Affiliations:** Department of Civil and Environmental Engineering, National University of Singapore, Singapore, 117576, Singapore; IPK, GERMANY

## Abstract

1,1,2-trichloroethane (1,1,2-TCA) has become a common groundwater pollutant due to historically extensive utilization, improper disposal, as well as from incomplete dechlorination of 1,1,2,2-tetrachloroethane. Currently, limited information is available on microbial detoxification of 1,1,2-TCA. *Desulfitobacterium* sp. strain PR, which was isolated from an anaerobic bioreactor maintained to dechlorinate chloroethenes/ethanes, exhibited the capacity to dechlorinate 1,1,1-trichloroethane and chloroform. In this study, the dechlorinating ability of strain PR was further explored. Strain PR showed the capability to dechlorinate 1,1,2-TCA (~1.12 mM) predominantly to 1,2-dichloroethane (1,2-DCA) and chloroethane, and to trace amounts of vinyl chloride and ethene within 20 days. Strain PR coupled growth with dechlorination of 1,1,2-TCA to 1,2-DCA, while no cell growth was observed with dechlorination of 1,2-DCA to chloroethane. Later, through transcriptomic and enzymatic analysis, the reductive dehalogenase CtrA, which was previously reported to be responsible for 1,1,1-trichloroethane and chloroform dechlorination, was identified as the 1,1,2-TCA reductive dehalogenase. Since trichloroethene (TCE) is usually co-contaminated with 1,1,2-TCA, a co-culture containing *Dehalococcoides mccartyi* strain 11a capable of detoxifying TCE and 1,2-DCA and strain PR was established. Interestingly, this co-culture dechlorinated 1,1,2-TCA and TCE to the non-toxic end-product ethene within 48 days without chloroethane production. This novel pathway avoids production of the carcinogenic intermediate dechlorination product vinyl chloride, providing a more environmentally friendly strategy to treat 1,1,2-TCA.

## Introduction

1,1,2-trichloroethane (1,1,2-TCA), molecular formula CHCl_2_-CH_2_Cl, has been widespread in the environment by extensive usage as a degreasing agent, through improper storage and disposal, and from incomplete dechlorination of the solvent 1,1,2,2-tetrachloroethane. 1,1,2-TCA is very persistent in the environment, with an estimated half-life of 136–360 days in soil and 136–720 days in groundwater [1]. It is found in at least 262 out of 1293 sites in the United States on the National Priorities List (NPL) identified by the U.S. Environmental Protection Agency [2]. The geometric mean maximum concentrations of 1,1,2-TCA is 0.57 μM in water and 2.14 mg/kg in soil [2]. Pollution caused by 1,1,2-TCA has raised concerns because of its potential for adverse effects on the liver, the kidneys, and the nervous and immune systems [1]. Detoxification of 1,1,2-TCA has historically been focused on chemical oxidation or physical adsorption [3, 4]. However, both of these approaches are limited by high energy and material costs, as well as the potential for incomplete degradation of 1,1,2-TCA.

Since the first report of anaerobic dechlorination of 1,1,2-TCA by a chlorobenzoate enriched bioreactor containing *Desulfomonile tiedjei* strain DCB-1 in 1994 [5], the promised potential of microorganisms for treatment of this environmental contaminant has been demonstrated (Table 1). Although the enriched culture containing strain DCB-1 exhibited the ability to dechlorinate 1,1,2-TCA, it failed to convert 63% of an initial concentration of ~10.7 μM 1,1,2-TCA to 1,2-DCA. Also, no experimental evidence was provided to support the involvement of *D*. *tiedjei* in the dechlorination process. The methanogen *Methanobacterium thermoautotrophicum* was also observed to cometabolically dechlorinate 1 μM of 1,1,2-TCA to vinyl chloride (89%), 1,2-DCA (11%), and a trace amount of ethane at 60°C in a complex medium [6]. Both uncharacterized mixed cultures [7, 8] and other reported strains belonging to the *Desulfitobacterium*, *Dehalobacter*, and *Dehalogenimonas* genera [9–13] have been shown to dechlorinate 1,1,2-TCA through dihaloelimination, resulting in the accumulation of vinyl chloride as the end product. Vinyl chloride is an identified carcinogenic compound and ranks 4^th^ on the U.S. EPA National Priorities List [2, 14]. Therefore, it is essential to look for alternative pathways that circumvent the production of vinyl chloride so as to achieve complete detoxification of 1,1,2-TCA. For 1,2-DCA, another metabolic intermediate of 1,1,2-TCA, is also a groundwater pollutant found in 582 of 1293 NPL sites in the United States identified by the U.S. Environmental Protection Agency [2]. Although 1,2-DCA is much less toxic than vinyl chloride, its removal is still of importance.

**Table 1:** Mixed cultures and isolates capable of anaerobically dechlorinating 1,1,2-TCA.

Sample ID	Initial conc. (μM)	Products	Sample nature	Metabolic Process	Ref.
*Desulfomonile tiediei* strain DCB-1	10.7^a^	1,2-DCA (Major)	Isolate	No	[5]
Unknown	16.2	VC(80%);1,2-DCA (20%); Ethane & ethane (trace amount)	Mixed culture	N/A^b^	[7]
*Methanobacterium thermoautotrophicum*	N/A	VC(89%);1,2-DCA(10%); Ethene (trace amount)	Isolate	No	[6]
*Desulfitobacterium dichloroeliminans* strain DCA1	N/A	VC	Isolate	Yes	[12]
Unknown	N/A	VC(Major)	Mixed culture	N/A	[8]
*Dehalobacter* sp.	~670	VC	Mixed culture	Yes	[11]
*Dehalogenimonas alkenigignens* strain IP3-3	~2420	VC	Isolate	Yes	[9]
*Dehalogenimonas lykanthroporepellens* strain BL-DC-9	~1650	VC	Isolate	Yes	[13]
*Dehalogenimonas alkeniginens* strain SBP-1	N/A	VC	Isolate	Yes	[9]
*Desulfitobacterium* sp. strain PR	~1400	1,2-DCA,CA, VC & Ethene (trace amount)	Isolate	Yes	This study

^a^ 1,1,2-TCA was not completely dechlorinated (only 37% conversion).

^b^Not available.

*Desulfitobacterium* sp. strain PR was isolated from a bioreactor performing reductive dechlorination of chloroethenes and chloroethanes [15]. This strain has been shown to reductively dechlorinate 1,1,1-TCA and chloroform, and harbours two reductive dehalogenase (RDase) genes, *ctrA* and *prdhA*. CtrA has been identified to reductively dechlorinate 1,1,1-TCA and chloroform, but the function of *prdhA* remains unknown. In this study, the dechlorination capability of strain PR was further explored on 1,1,2-TCA and the responsible 1,1,2-TCA RDase gene was identified by both gene expression studies and proteomics tools. Due to the accumulation of 1,2-DCA from dechlorination of 1,1,2-TCA by strain PR, a *Dehalococcoides*-containing mixed culture GEO, which was enriched from the same source as strain PR and dechlorinates 1,2-DCA rapidly, was co-cultivated with strain PR to explore potential for complete detoxification of 1,1,2-TCA. Additionally, to achieve complete detoxification of 1,1,2-TCA and to address the problems posed by co-contamination of trichloroethene (TCE) and 1,1,2-TCA, a co-culture consisting of strain PR and *Dehalococcoides mccartyi* strain 11a was established. *Dehalococcoides mccartyi* strain 11a was chosen to address co-contamination caused by TCE and 1,1,2-TCA since it can reductively dechlorinate both TCE and 1,2-DCA [16].

## Materials and Methods

### Chemicals and bacterial growth conditions

All chemical reagents including components in DCB1 medium, chlorinated chemicals and reagents used in *in vitro* assays were purchased from Sigma-Aldrich (St. Louis, MO) or Merck (Darmstadt, Germany) at the highest purity available. Cultures were grown in an anaerobic bicarbonate-buffered defined DCB1 medium. Strain PR and culture GEO were spiked with filter-sterilized pyruvate (10 mM) as the carbon source/electron donor and 1,1,2-TCA, 1,1,1-TCA or chloroform as the electron acceptor. *Dehalococcoides mccartyi* strain 11a was spiked with sodium acetate (10 mM) as the carbon source, hydrogen as the electron donor (0.33 atm) and TCE as the electron acceptor as previously described [17, 18]. For all dechlorination kinetics studies and cell collections for proteomic analysis, strain PR was grown in 160-mL serum bottles containing 100 mL medium at 30°C. TCE ranging from 0.002 mM to 1.12 mM were added to cultures provided with 0.30 mM 1,1,2-TCA to explore TCE inhibitory effects on 1,1,2-TCA dechlorination. Cell density of cultures amended with 1,1,2-TCA ranging from 0.10 mM to 1.20 mM at day 4 was used to assess net growth yield per moles of 1,1,2-TCA. Two sets of experiments were conducted to investigate the cometabolic dechlorination of 1,2-DCA to chloroethane. In the first set, 0.92 mM 1,2-DCA was added as the sole electron acceptor. In the second set, 0.30 mM 1,1,2-TCA was spiked initially, and another 0.30 mM 1,1,2-TCA was spiked on day 8. When exploring dechlorinating preference of strain PR on different substrates, 0.51 mM 1,1,2-TCA and 0.59 mM chloroform was amended simultaneously. All kinetics studies were performed in triplicate with abiotic controls (without bacterial inocula or with autoclaved cultures).

### Sample analytical procedures

Headspace samples of chloromethanes, chloroethanes, and chloroethenes were analyzed with an Agilent gas chromatograph (GC7890) equipped with a flame ionization detector and a GS-GasPro column (30 m by 0.32 mm; J&W Scientific). The oven temperature was initially held at 50°C for 2 min, increased to 220°C at 30°C /min and held for 1min. Headspace samples were manually injected to the GC using a gas-tight glass syringe (Model Gastight #1725, Hamilton Co., Reno, Nevada).

### Molecular analyses

Cells for DNA extraction were collected from 1 mL culture samples by centrifugation (10 min at 10,000 g, 4°C). Cell pellets were stored at -20°C until further processing. Genomic DNA was extracted using the Qiagen DNeasy Blood and Tissue Kit (QIAGEN GmbH, Hilden, Germany) according to the manufacturer’s instructions. Quantitative real-time PCR (qPCR) was conducted on an ABI 7500 Fast System using SYBR green (Lo-ROX, SensiFAST SYBR, BIOLINE) as described previously [19]. The thermocycling program was as follows: an initial step of 3 min at 95°C, followed by 40 cycles of 5 s at 95°C and 30s at 60°C. Construction of plasmids carrying targeted genes to serve as qPCR standards was performed as described previously [20]. *Desulfitobacterium*-genus specific primers (Dsb1299F and Dsb1448R), *ctrA* gene primers (ctrAF and ctrAR), and *prdhA* gene primers (prdhAF and prdhAR) were utilized to monitor cell growth and gene expression levels in strain PR [15]. To identify the 1,1,2-TCA RDase genes, transcription of *ctrA* and *prdhA* were monitored during dechlorination of 1,1,2-TCA. 5% (v) inoculum (starved for 3 days) was transferred to fresh medium with or without 1,1,2-TCA. Duplicate cell samples were collected at 12 hour intervals for DNA (1 mL of culture) and RNA (1.5 mL of culture) extractions (10 min at 10,000 g, 4°C). The RNA extraction and reverse transcription were conducted as described previously [15]. That is, Trizol was used for cell lysis and RNA stabilization. The QIAGEN RNeasy mini kit was used for RNA extraction (QIAGEN GmbH) according to the manufacturer’s instructions. Luciferase control RNA (Promega, Fitchburg, WI, USA) was added before RNA extraction as an internal standard to evaluate mRNA loss during RNA extraction, reverse transcription and quantification. Reverse transcription was conducted directly after RNA elution using the QIAGEN Sensiscript kit according to the manufacturer’s instructions with random hexa primer (Promega) and RNase inhibitor (Promega).

### Enzyme assays

To characterize the localization of 1,1,2-TCA RDase, membrane and crude proteins each collected from 100 mL cultures were used for *in vitro* activity assays. Cell pellets were collected by centrifugation at 10,000 g for 20 min at 4°C and re-suspended in 0.1 mL fresh DCB1 medium. To prepare crude proteins, cells were broken by ultrasonication using a VCX130 sonicator (Vibra-Cell, SONICS) (130W; 20% duty cycle; for 3 min). The lysate was centrifuged at 10,000 g for 30 min and the supernatant containing the crude protein was harvested. To obtain membrane proteins, the crude protein extract was ultracentrifuged at 120,000 g for 1 h at 4°C. The pellets containing membrane proteins were solubilized in 10 mM CHAPS and concentrated by centrifugation through a membrane filter with a molecular cutoff of 5 kDa (Vivaspin2, Sartorius Stedium Biotech). The *in vitro* activity assay was carried out in 10-mL vials containing 2 mL assay solution (2 mM methyl viologen; 1.5 mM titanium (III) citrate; 100 mM Tris-HCl buffer (pH 7.0)) spiked with 300 μM 1,1,2-TCA inside an anaerobic chamber as described previously [19, 21]. The mixtures were incubated at 30°C for 72 h prior to GC analysis.

1,1,2-TCA RDase substrate range characterization was performed with 0.1 mM of each substrate (1,1,2-TCA, 1,1,1-TCA, 1,2-DCA, 1,1-DCA or chloroform) added to duplicate 4-mL vials containing the assay solution. Crude extracts were obtained by disrupting harvested cells with a VCX 130 sonicator (Vibra-Cell, SONICS) (130W; 20% duty cycle; for 3 min). Crude extracts obtained from cultures grown with 1,1,2-TCA were added to 2 mL assay solution. The mixtures were incubated at 30°C for 24 h prior to headspace analysis. The effect of substrate concentration on the rate of dechlorination for each substrate (1,1,1-TCA, 1,1,2-TCA, or chloroform) was assayed. For each substrate, crude cell extracts from cultures grown on the corresponding substrate was incubated at 30°C for 2 h with six concentrations of substrates ranging from 0.05 mM to 2.50 mM. Reactions were quenched by adding 0.5 mL of hydrochloric acid solution (0.012 mM) and analyzed by GC. All enzyme assays were performed with abiotic controls without addition of crude cell extract.

### Proteomic analyses

Native polyacrylamide gels (native-PAGE) with a 10% resolving gel and a 5% stacking gel were prepared as described previously [22], except that the experiment was carried out in an ice box and sodium dodecyl sulfate (SDS) was removed from all buffers to avoid protein denaturation.

Native PAGE analysis using a modified staining protocol was conducted with crude cell proteins [21]. Briefly, the same protein samples were added on adjacent gel lanes. Gel electrophoresis was run at 160 V for 2 h. After electrophoresis, one lane was silver stained using the PlusOne Silver Staining kit according to manufacturer’s instructions, while the other lane was left unstained. Bands in the unstained lane were excised for RDase activity assays with 0.05 mM of substrate (1,1,2-TCA, 1,1,1-TCA, or chloroform). After 24 h of incubation at 30°C, headspace of each sample was analyzed by GC.

Prominent bands in the stained lanes corresponding to gel regions exhibiting 1,1,2-TCA dechlorinating activity in non-stained bands were cut and analyzed by MALDI-TOF-MS at the Protein and Proteomics Centre, National University of Singapore. Gel bands were trypsin digested and loaded onto an ABI Voyager STR MALDI-TOF mass spectrometer. Output signals were searched for probable protein hits in the Mascot database, which was constructed based on the National Center for Biotechnology Information Database.

## Results

### Growth coupling dechlorination of 1,1,2-TCA

**Fig 1:**
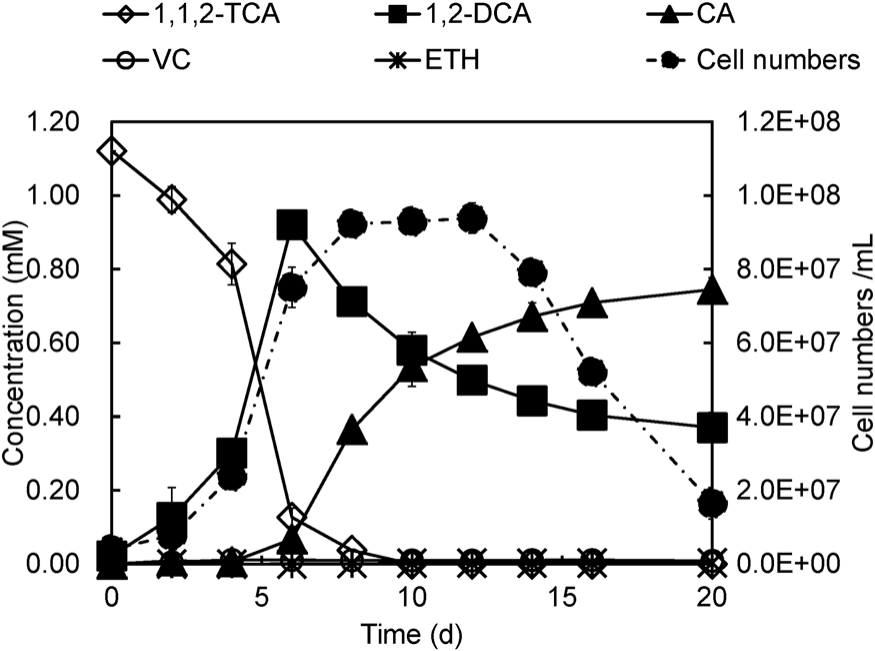
Dechlorination of 1,1,2-TCA by *Desulfitobacterium* sp. strain PR. Note: CA, chloroethane; VC, vinyl chloride; ETH, ethene.

In contrast to bacteria that dechlorinate 1,1,2-TCA to vinyl chloride via dihaloelimination, strain PR completely dechlorinated 1,1,2-TCA (~1.12 mM) to 1,2-DCA and chloroethane at a 1: 2 ratio within 20 days via reductive dechlorination (Fig. 1). The concentration of 1,2-DCA reached the highest point (0.92 mM) on day 6 and dropped to 0.37 mM at the end of the dechlorination process. Chloroethane started to accumulate on day 4, reaching a final concentration of 0.75 mM on day 20. Only trace amounts (~0.001 mM) of vinyl chloride and ethene were produced. No 1,1,2-TCA dechlorination was observed in the abiotic controls (neither in cultures without inocula nor in autoclaved cultures). Strain PR dechlorinated 1.12 mM of 1,1,2-TCA (CH_2_Cl-CHCl_2_) within 10 days (from day 0 to day 10) at an average dechlorination rate of 0.11 mM/day, while 0.55 mM of 1,2-DCA (CH_2_Cl-CH_2_Cl) was dechlorinated to chloroethane within 14 days (from day 6 to day 20) at an average dechlorination rate of 0.028 mM/day. The average dechlorination rate of the dihalomethyl (-CCl_2_) group is almost four times higher than that of the monohalomethyl (-CCl) group. During dechlorination of 1,1,2-TCA to 1,2-DCA, cell growth was closely coupled to the consumed amount of 1,1,2-TCA. Regression between cell numbers and the amount of chlorine removal shows a linear correlation, indicating a growth yield of ~3.72 × 10^13^ cells / mole chlorine removed (S1 Fig.). Together, these phenomena implicate 1,1,2-TCA as a growth-supporting electron acceptor for strain PR; no further cell growth was observed after 1,1,2-TCA was depleted on day 8. However, later experiments showed that cell growth occurred upon supplementary addition of 1,1,2-TCA after the initial 1,1,2-TCA had been depleted (S2 Fig.). Additionally, no 1,2-DCA dechlorination was observed when 1,2-DCA was fed to strain PR as the sole electron acceptor. These results indicate that dechlorination of 1,2-DCA to chloroethane is a cometabolic process.

**Fig 2:**
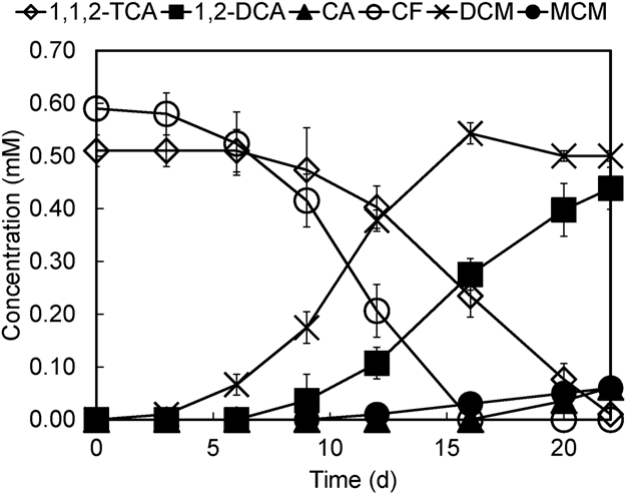
Dechlorination of 1,1,2-TCA and chloroform simultaneously by strain PR. Note: CA, chloroethane; DCM, dichloromethane; MCM, chloromethane.

To understand the substrate preference of strain PR on 1,1,2-TCA and chloroform, dechlorinating profile was monitored in cultures amended with 1,1,2-TCA and chloroform simultaneously. Strain PR exhibited a longer lag phase to dechlorinate 1,1,2-TCA as compared to chloroform (Fig. 2). Dechlorination of 1,1,2-TCA occurred more slowly than that of chloroform, an average rate of 0.036 mM per day (day 6 to day 22) versus 0.045 mM per day (day 3 to day 16), and did not commence until ~16% of the chloroform had been converted to dichloromethane.

### Identification of 1,1,2-TCA RDase gene ctrA in strain PR

**Fig 3:**
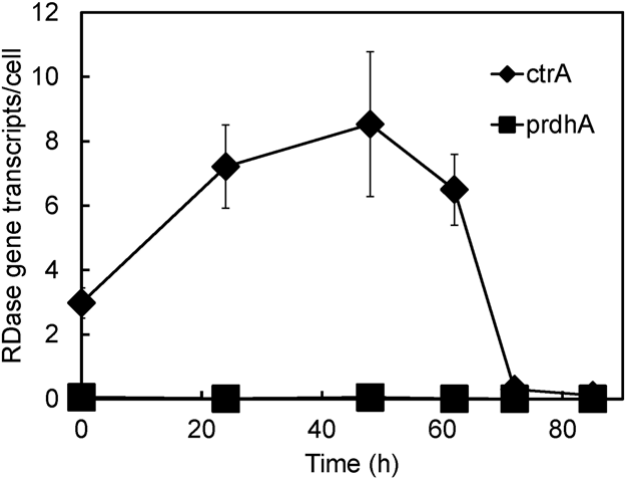
Transcription of *ctrA* and *prdhA* genes in *Desulfitobacterium* sp. strain PR fed with 1,1,2-TCA.

Strain PR harbours both a functionally characterized RDases gene, *ctrA*, and an as-yet uncharacterized RDase gene, *prdhA* [15]. In order to determine whether these genes are responsible for 1,1,2-TCA dechlorination in strain PR, transcripts of *ctrA* and *prdhA* were monitored during exposure to 1,1,2-TCA. As shown in Fig. 3, transcription of the *ctrA* gene increased to 8.53 transcripts/cell prior to the onset of 1,1,2-TCA dechlorination, while the *prdhA* gene showed negligible transcription.

**Fig 4:**
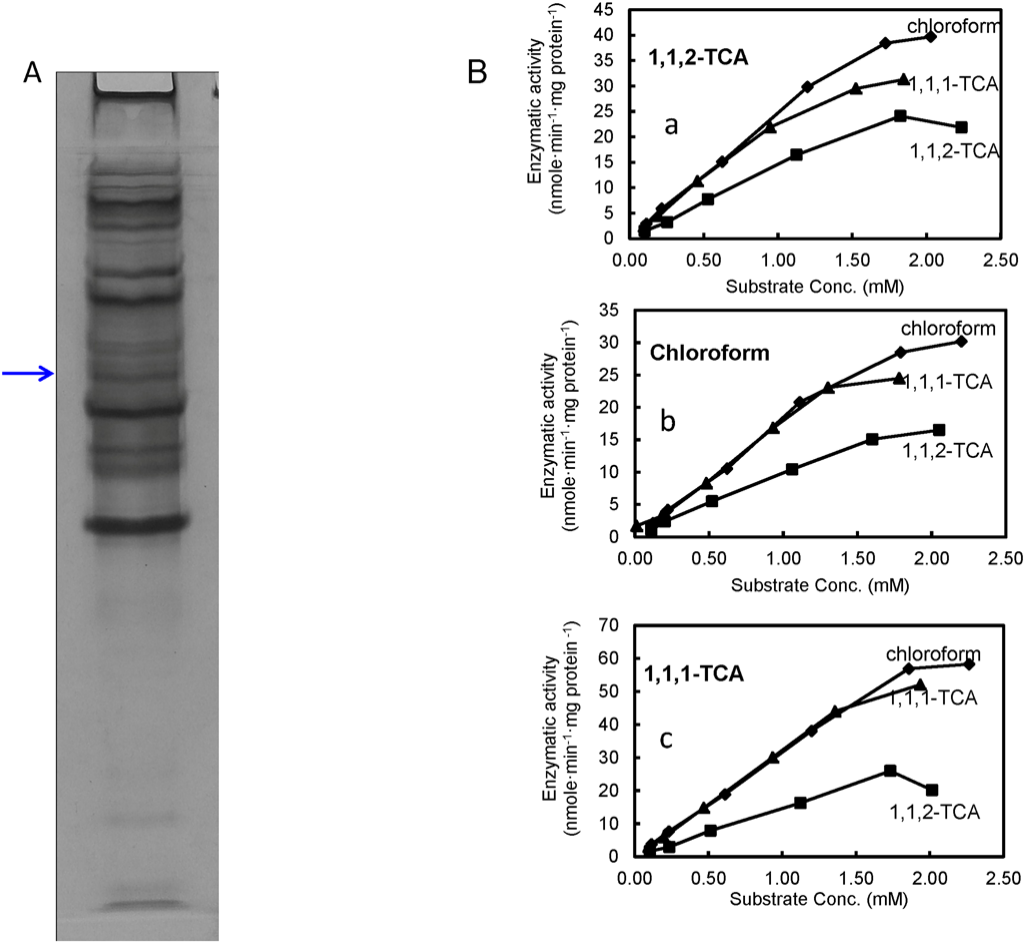
Identification and characterization of 1,1,2-TCA RDase. (**A**) Native-PAGE gel profile of crude cell proteins of strain PR fed with 1,1,2-TCA. **(B)** Dechlorination rates of crude cell extracts upon exposure to various concentrations of 1,1,2-TCA,1,1,1-TCA, or chloroform. The cell extracts were obtained from culture PR fed with (a) 1,1,2-TCA, (b) 1,1,1-TCA, or (c) chloroform.

The role of CtrA in 1,1,2-TCA dechlorination was further confirmed by native-PAGE. Crude cell extracts taken from strain PR during exponential growth phase were separated on a native PAGE gel and dechlorinating activity was recovered directly from native gels. The corresponding dechlorination products (1,2-DCA, 1,1-DCA, or dichloromethane) from 1,1,2-TCA, 1,1,1-TCA and chloroform were detected from the same single band, while other gel fragments failed to show dechlorinating activity. This indicates that the proteins in this band are responsible for dechlorination of the above-mentioned chlorinated compounds. The silver stained counterpart of the gel fragment showing dechlorination activity was analyzed by MALDI-TOF-MS (Fig. 4A). Results from MALDI-TOF-MS analysis identified CtrA as the only detected reductive dehalogenase in the active gel fragment (Mascot score of 447 with 41.8% sequence coverage). Together, these analyses strongly implicate the *ctrA* gene as responsible for dechlorinating 1,1,2-TCA as well as 1,1,1-TCA and chloroform in strain PR.

### Characterization of CtrA gene in strain PR

Both crude proteins and membrane proteins from strain PR showed identical dechlorination profiles for 1,1,2-TCA. This suggests that 1,1,2-TCA dechlorination was mediated by a membrane-associated protein. Substrate range characterization revealed that the CtrA in strain PR produced the metabolites 1,2-DCA, 1,1-DCA, dichloromethane and chloroethane from 1,1,2-TCA, 1,1,1-TCA, chloroform and DCAs, respectively. Similar to dechlorination performance in active cultures of strain PR, dechlorination of 1,2-DCA to chloroethane was slow and only a small amount of 1,2-DCA had been dechlorinated when most TCAs were dechlorinated to DCAs. Induction of the *ctrA* gene by 1,1,1-TCA and chloroform yielded identical *in vitro* enzyme kinetics.

Fig. 4B shows the effect of substrate concentration on the rate of dechlorination for each substrate (1,1,1-TCA, 1,1,2-TCA, or chloroform) by using crude cell extracts. The dechlorination rates increased linearly as the initial electron acceptor concentrations increased, and the dechlorinate rates ceased to increase as electron acceptor concentrations reached around 2.00 mM. The maximum reaction rates, *v*_*max*,_ for each substrate are in the same order of magnitude ranging from 15 to 60 nmoles substrate dechlorinated per min per mg protein. Interestingly, the dechlorination rates of 1,1,1-TCA and chloroform were ~1.5–2.0 times that of 1,1,2-TCA.

### Establishment of co-cultures for complete dechlorination of 1,1,2-TCA

**Fig 5:**
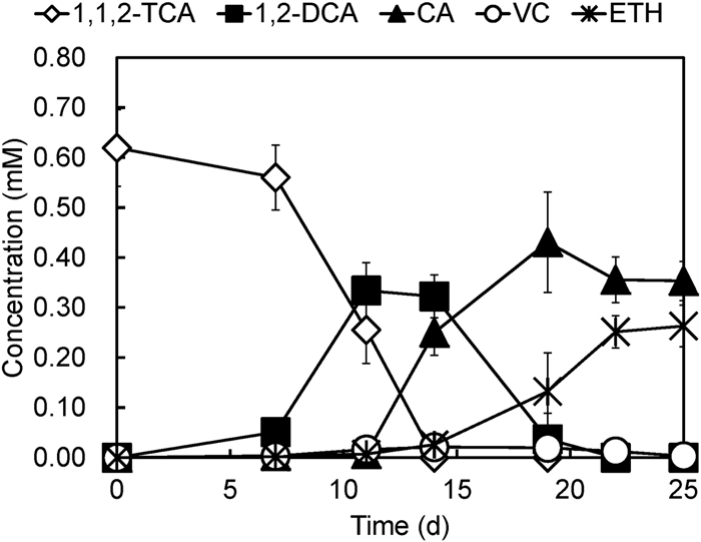
Dechlorination of 1,1,2-TCA by a co-culture consisting of strain PR and a mixed culture GEO. Note: CA, chloroethane; VC, vinyl chloride; ETH, ethene.

In order to achieve complete dechlorination of 1,1,2-TCA, a co-culture was established with strain PR and a highly enriched mixed culture, GEO, that is capable of dechlorinating 1,2-DCA to ethene via dihaloelimination. When 1,1,2-TCA (~ 0.62 mM) was spiked into the medium, this co-culture sequentially dechlorinated 1,1,2-TCA to 1,2-DCA and chloroethane on day 11 (Fig. 5). Most of the 1,2-DCA produced was rapidly dechlorinated to ethene, however, a fraction of 1,2-DCA was still dechlorinated to chloroethane co-metabolically by strain PR. By day 25, all of the 1,1,2-TCA had been converted to chloroethane (~ 0.35 mM) and ethene (~ 0.26 mM).

**Fig 6:**
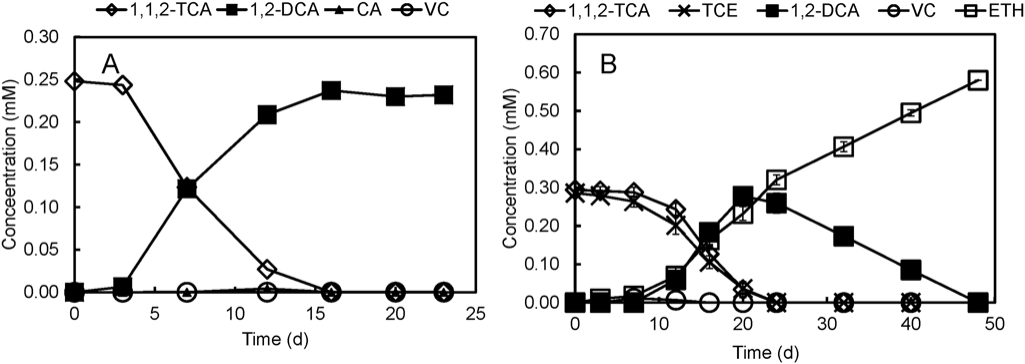
Co-culturing strain PR and *Dehalococcoides mccartyi* strain 11a on 1,1,2-TCA/TCE. (**A**) Inhibitory effects of 0.05 mM TCE on 1,1,2-TCA dechlorination by strain PR. (**B**) Dechlorination of 1,1,2-TCA and TCE by a co-culture consisting of strains PR and 11a. Note: CA, chloroethane; VC, vinyl chloride; ETH, ethene.

As a co-contaminant with 1,1,2-TCA, TCE ranging from 0.01 mM to 1.12 mM has no inhibitory effect on dechlorination of 1,1,2-TCA to 1,2-DCA by strain PR and S3 Fig. shows dechlorination profile at a representative low (0.12 mM) and high (0.24 mM) TCE concentration. Moreover, production of chloroethane by strain PR is inhibited by as little as 0.05 mM TCE in the medium, resulting in the production of only 1,2-DCA from the dechlorination of 1,1,2-TCA (Fig. 6A). In order to achieve complete dechlorination of 1,1,2-TCA without accumulation of either 1,2-DCA or chloroethane in the presence of TCE/1,1,2-TCA co-contaminants, another co-culture was established with strain PR and *Dehalococcoides mccartyi* strain 11a. After spiking TCE (0.29 mM) and 1,1,2-TCA (0.29 mM) to the co-culture, 1,1,2-TCA and TCE were dechlorinated at similar rates to 1,2-DCA (by strain PR) and ethene (by strain 11a), within 24 days (Fig. 6B). The amount of 1,2-DCA peaked (0.28 mM) on day 20, after which it was completely dechlorinated to ethene by strain 11a. Throughout the experiment, the concentration of CA was below detection limit (0.006 mM). A trace amount of VC (0.012 mM) was generated on day 7, but disappeared after 9 days. Overall, the co-culture of strains PR and 11a was able to achieve complete detoxification of 1,1,2-TCA and TCE to ethene.

## Discussion

Anaerobic bacterial dechlorination of 1,1,2-TCA by dihaloelimination, which results in the accumulation of the carcinogenic compound vinyl chloride, has been known since the 1990s [6–12]. In this study, we show that *Desulfitobacterium* sp. strain PR reductively dechlorinates 1,1,2-TCA to 1,2-DCA and chloroethane via a stepwise reductive dechlorination process. Strain PR metabolically dechlorinates a much higher concentration (1.12 mM versus 0.011mM) of 1,1,2-TCA than the previously reported cometabolic reductive dechlorination of 1,1,2-TCA to DCA by *Desulfomonile tiedjei* strain DCB-1 [5]. The growth yield of strain PR from dechlorination of 1,1,2-TCA is 3.72×10^13^ cells per mole chlorine, which is comparable to the growth yields from dechlorination of chloroform or 1,1,1-TCA by strain PR and of chloroethanes by other organohalide respiring bacteria [11, 15]. Strain PR exhibits a broad substrate range of chlorinated ethanes/methanes, including 1,1,2-TCA, 1,1,1-TCA and chloroform. Although structurally distinct, 1,1,2-TCA (-CCl_2_), chloroform (-CCl_3_), and 1,1,1-TCA (-CCl_3_) share similar functional groups (tri- or dichloromethyl groups). Unlike many other organohalide respiring bacteria that harbor multiple functional RDase genes which catalyze various substrates, such as *Dehalococcoides mccartyi* strains 195 and CBDB1 [23, 24], the *ctrA* gene in strain PR is responsible for dechlorination of 1,1,2-TCA, chloroform and 1,1,1-TCA. This finding suggests that organohalide respiring bacteria are able to reductively dechlorinate multiple substrates with similar functional groups by using a single RDase, which suggests that structural analogs should be considered during the exploration of potential substrate range in novel RDases. The two-fold difference in dechlorination rates exhibited by CtrA in enzyme assays in catalysis of the trihalomethyl (-CCl3) and the dihalomethyl (-CCl2-) groups suggests that a higher number of chlorine substitutes could lead to higher dechlorination rates for CtrA in strain PR. Similarly, when 1,1,2-TCA and chloroform co-exist, strain PR exhibits a faster rate of dechlorination for chloroform than for 1,1,2-TCA. These findings agree with previous reports indicating a relationship between chlorine number and dechlorination rate [25].

Enzyme CtrA shows a slight substrate binding preference to chloroform in the presence of chloroform and 1,1,2-TCA. Also, the cometabolic dechlorination from 1,2-DCA to chloroethane is not affected by the presence of 1,1,2-TCA or chloroform. TCE inhibits the conversion of 1,2-DCA to chloroethane, but not that of 1,1,2-DCA to 1,2-DCA. The complete dechlorination from 1,1,2-TCA to 1,2-DCA shows that TCE does not inhibit CtrA. Therefore, the cessation of 1,2-DCA dechlorination might result from competitive inhibition by TCE over 1,2-DCA. In all, CtrA demonstrates a substrate binding preference as: chloroform (CCl3-H) > 1,1,2-TCA (H-CCl2-CCl-H2) >> TCE (CCl2 = CCl2) >> 1,2-DCA (H2-CCl-CCl-H2).

Chloroethane was accumulated in the co-culture comprising strain PR and the mixed culture GEO amended solely with 1,1,2-TCA. Although the same dechlorinating capability (1,2-DCA to ethene) is shared by mixed culture GEO and strain 11a, complete detoxification of TCE/1,1,2-TCA by co-culturing strain PR and 11a was achieved after the additional amendment of TCE. TCE inhibited the dechlorination of 1,2-DCA to CA, effectively eliminating the accumulation of chloroethane. In the presence of TCE/1,1,2-TCA and co-cultivated with strain PR, strain 11a started to dechlorinate 1,2-DCA when the initial TCE was depleted, indicating that strain 11a preferentially dechlorinates TCE over 1,2-DCA when both are available. This may be a result of the ~ 5 times faster dechlorination rate of TCE than that of 1,2-DCA by strain 11a, although both TCE and 1,2-DCA are likely catalyzed by the same RDase gene, *vcrA*, in strain 11a [16].

In summary, this study is the first report of metabolic reductive dechlorination of 1,1,2-TCA to chloroethanes and of identification of the enzyme responsible for this process. By co-culturing strain PR with *Dehalococcoides mccartyi* strain 11a, 1,1,2-TCA and TCE can be completely dechlorinated to a non-toxic end product, ethene. The characterized properties of strain PR enlarge our view of its versatile dechlorinating performance, making it a promising candidate for broad applications in *in situ* bioremediation.

## Supporting Information

S1 Fig Linear correlation between cell number of strain PR and chlorine removed from 1,1,2-trichloroethane on day 4. (TIF)

S2 Fig Dechlorination of 1,1,2-TCA by *Desulfitobacterium* sp. strain PR via stepwise spiking 1,1,2-TCA. Note: CA, chloroethane.
(TIF)

S3 Fig Inhibition effects of TCE (A) 0.12 mM and (B) 0.24 mM on 1,1,2-TCA dechlorination activity by strain PR. Co-culture was cultivated in 30 mL serum bottles, amended with acetate and pyruvate (10 mM each) together with H_2_ (10 mL). Strain PR was inoculated on day 0, while strain 11a was inoculated on day 13. Note: CA, chloroethane; VC, vinyl chloride; ETH, ethene.
(TIF)
