# Supplementary figures and images for: Detoxification of 1,1,2-Trichloroethane to Ethene by Desulfitobacterium and Identification of Its Functional Reductase Gene
Source: PLoS One. 2015 Apr 2;10(4):e0119507. doi: 10.1371/journal.pone.0119507 (PMC4383557; doi:10.1371/journal.pone.0119507)

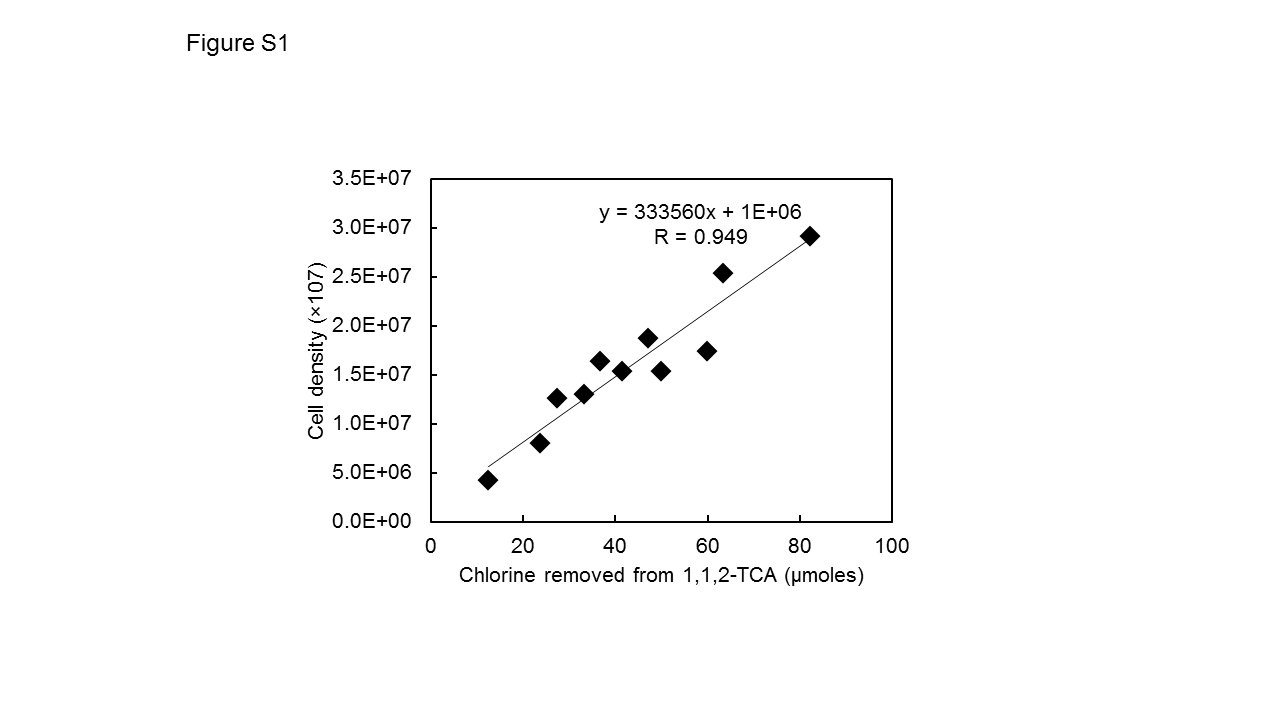

Supplement: S1 Fig — (TIF) [file pone.0119507.s001.TIF]

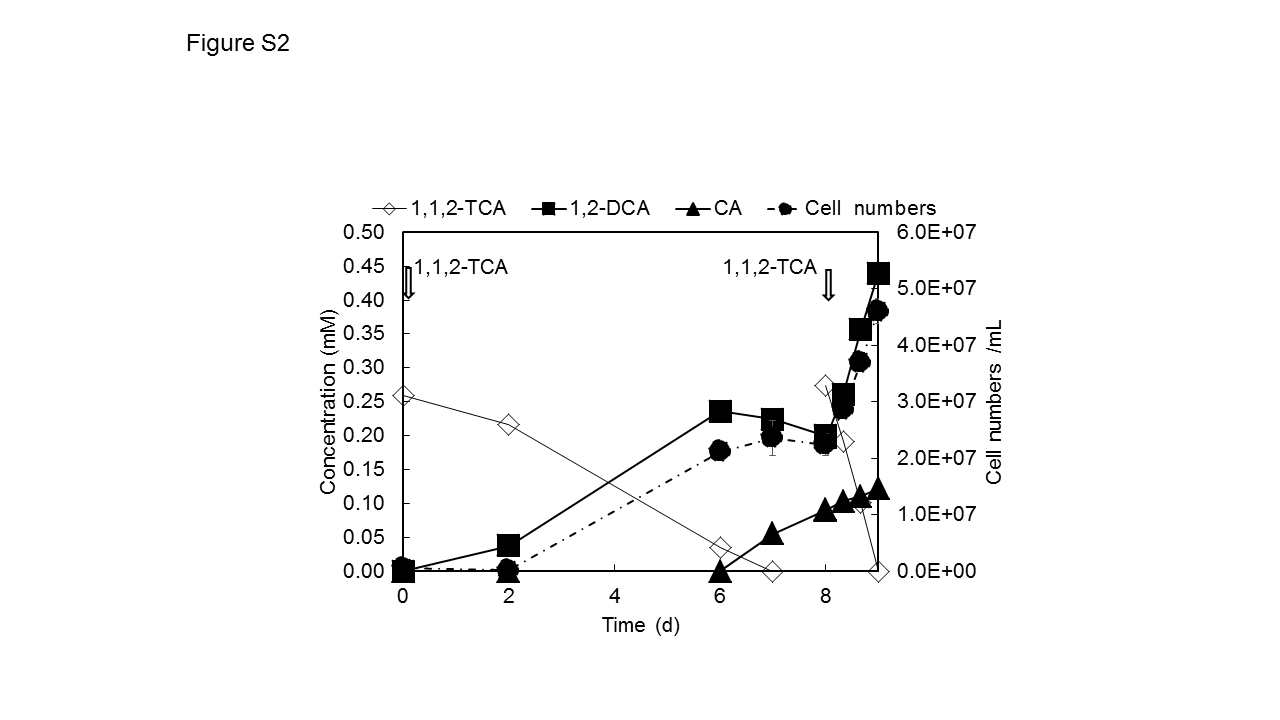

Supplement: S2 Fig — Note: CA, chloroethane. (TIF) [file pone.0119507.s002.TIF]

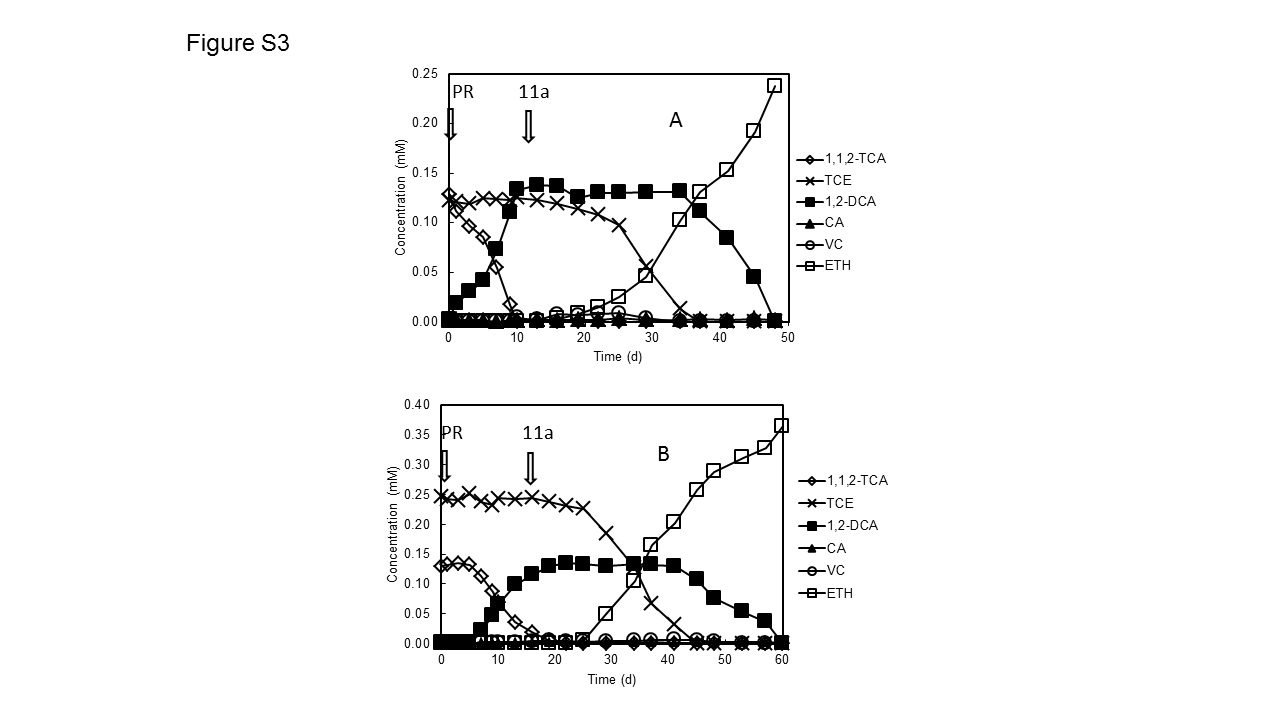

Supplement: S3 Fig — Co-culture was cultivated in 30 mL serum bottles, amended with acetate and pyruvate (10 mM each) together with H2 (10 mL). Strain PR was inoculated on day 0, while strain 11a was inoculated on day 13. Note: CA, chloroethane; VC, vinyl chloride; ETH, ethene. (TIF) [file pone.0119507.s003.TIF]
